# *Bacteroides* and NAFLD: pathophysiology and therapy

**DOI:** 10.3389/fmicb.2024.1288856

**Published:** 2024-03-20

**Authors:** Jun Zhang, Jing Zhou, Zheyun He, Hongshan Li

**Affiliations:** ^1^Liver Disease Department of Integrative Medicine, Ningbo No. 2 Hospital, Ningbo, Zhejiang, China; ^2^Cixi Biomedical Research Institute, Wenzhou Medical University, Ningbo, Zhejiang, China; ^3^Liver Diseases Institute, Ningbo No. 2 Hospital, Ningbo, Zhejiang, China; ^4^Key Laboratory of Diagnosis and Treatment of Digestive System Tumors of Zhejiang Province, Ningbo, Zhejiang, China

**Keywords:** *Bacteroides*, NAFLD, steatosis, liver inflammation, intestinal barrier

## Abstract

Non-alcoholic fatty liver disease (NAFLD) is a prevalent chronic liver condition observed globally, with the potential to progress to non-alcoholic steatohepatitis (NASH), cirrhosis, and even hepatocellular carcinoma. Currently, the US Food and Drug Administration (FDA) has not approved any drugs for the treatment of NAFLD. NAFLD is characterized by histopathological abnormalities in the liver, such as lipid accumulation, steatosis, hepatic balloon degeneration, and inflammation. Dysbiosis of the gut microbiota and its metabolites significantly contribute to the initiation and advancement of NAFLD. *Bacteroides*, a potential probiotic, has shown strong potential in preventing the onset and progression of NAFLD. However, the precise mechanism by which *Bacteroides* treats NAFLD remains uncertain. In this review, we explore the current understanding of the role of *Bacteroides* and its metabolites in the treatment of NAFLD, focusing on their ability to reduce liver inflammation, mitigate hepatic steatosis, and enhance intestinal barrier function. Additionally, we summarize how *Bacteroides* alleviates pathological changes by restoring the metabolism, improving insulin resistance, regulating cytokines, and promoting tight-junctions. A deeper comprehension of the mechanisms through which *Bacteroides* is involved in the pathogenesis of NAFLD should aid the development of innovative drugs targeting NAFLD.

## Introduction

With the global surge in obesity, there has been a parallel increase in diseases related to obesity, such as type 2 diabetes mellitus (T2DM), fatty liver disease, and cardiovascular disease (Badmus et al., 2022). Non-alcoholic fatty liver disease (NAFLD) is characterized by the excessive accumulation of intrahepatic fat, known as hepatic steatosis, in the absence of heavy alcohol abuse (Hrncir et al., 2021). NAFLD covers a spectrum of diseases, ranging from non-alcoholic hepatic steatosis to non-alcoholic steatohepatitis (NASH), and in severe cases, advanced cirrhosis and hepatocellular carcinoma (Kumar et al., 2020). The incidence and prevalence of NAFLD have surged globally in recent years, mirroring the rise in the obese population. In 1990–2019, NAFLD affected approximately 30% of the global population, with regional variations ranging from 25% in Western Europe to 44% in Latin America (Younossi et al., 2023). Mounting evidence suggests that NAFLD is closely associated with metabolic diseases and cardiovascular diseases, including obesity, T2DM, hypertriglyceridemia, and hypertension (Lonardo et al., 2018). With its escalating morbidity and mortality rates, NAFLD has emerged as a major chronic liver disease also in China, imposing a substantial economic burden on society and necessitating extensive further research to understand and tackle it (Chen H. et al., 2022).

Non-alcoholic fatty liver disease (NAFLD) is recognized as a multifactorial disease resulting from complex interactions between genetic and environmental factors, with its pathogenesis not yet fully understood (Bibbo et al., 2018). Initially, the “two-hit” theory was proposed to explain the development of NAFLD (Zhang et al., 2018). However, this theory has been deemed outdated by some researchers, who instead advocate for the “multiple-hit” theory, positing that NAFLD involves various mechanisms influenced by the host susceptibility, environment, genetics, and gut microbiota. The concept of the gut–liver axis has garnered increasing attention, with research highlighting the intricate relationship between the intestine and liver diseases (Paolella et al., 2014). A recent study linked the pathogenesis of NAFLD to alterations in the gut microbiota (Lee et al., 2021). This view was reinforced by Hoyles et al. (2018), who observed that the transplantation of gut microbiota from NAFLD patients into germ-free mice led to hepatic steatosis and the emergence of gut microbiota with similar characteristics to those found in NAFLD patients, suggesting that the gut microbiota play a pivotal role in the development of NAFLD.

Probiotics play a crucial role in maintaining or restoring the homeostasis of gut microbiota, representing a key factor in alleviating NAFLD and preserving the host’s overall health status (Kaufmann et al., 2023). Among the identified probiotics, *Lactobacillus* and *Akkermansia* have demonstrated the ability to mitigate liver damage, metabolic abnormalities in the host, and disruptions in the gut microbiota caused by a high-fat diet (HFD) (Cani and de Vos, 2017; Arellano-Garcia et al., 2023). However, the cultivation of these two species is challenging, and their therapeutic efficacy may be influenced by environmental factors. In contrast, *Bacteroides*, as potentially beneficial organisms, exhibit greater stability and provide energy to adjacent bacteria (Zafar and Saier, 2021). Consequently, *Bacteroides* has emerged as a promising probiotic (Tan et al., 2019a).

*Bacteroides*, the predominant genus in the *Bacteroidetes* phylum, comprise Gram-negative bacteria that exhibit a rod-shaped morphology. They are characterized by their anaerobic nature, resistance to bile, lack of motility, and inability to form spores (Fernandez-Julia et al., 2021; Béchon and Ghigo, 2022). *Bacteroides* display rapid growth in diverse complex media, with chopped meats being the primary medium for their growth and storage. Notably, *Bacteroides* colonies possess a distinct smooth and rounded morphology, exhibiting frosted surfaces that range in color from a dull white to light beige (Bacic and Smith, 2008). *Bacteroides fragilis* (*B. fragilis*), a prevalent member of the *Bacteroides* genus, is commonly found in the human intestinal tract. When cultivated on *Bacteroides* bile esculin medium, *B. fragilis* hydrolyzes esculin, resulting in the formation of characteristic black colonies (Sun et al., 2019). It is important to note that *Bacteroides* do not undergo cell division in the presence of oxygen. Therefore, the key consideration in culturing *Bacteroides* lies in maintaining an anaerobic atmosphere (Bacic and Smith, 2008). Due to their ability to tolerate oxygen, ease of manipulation on laboratory benches, and relatively short generation time, *Bacteroides* serve as an ideal microbiota for investigating bacteria–host interactions (Béchon and Ghigo, 2022).

In recent years, researchers have proposed new treatments for NAFLD, such as bariatric surgery, endoscopic therapy, glucagon-like peptide 1 (GLP-1) receptor agonists, statins, vitamin E and fecal microbiota transplantation (Smits et al., 2013; Nguyen and Varela, 2017; Hadi et al., 2018; Azemawah et al., 2019; Doumas et al., 2019; Lefere et al., 2021; Mandour et al., 2022; Geerts and Lefere, 2023; Lee and Kim, 2023; Igi et al., 2024). Compared to these treatments, *Bacteroides*, a single-strain probiotic supplementation, is not only safe but also regulates the gut microbiota and treats the root cause of NAFLD (Carpi et al., 2022). We summarize the advantages and disadvantages of these treatment modalities in Table 1. Thus, *Bacteroides* have received increasing attention as it is now believed they may play a critical role in regulating hepatic metabolic processes. However, no review has yet summarized the interactions between *Bacteroides* and NAFLD. Consequently, in the present review, we elucidate the potential mechanisms underlying the therapeutic effects of *Bacteroides*, a major gut microbiota, in the treatment of NAFLD. This review offers a fresh perspective for NAFLD management.

**TABLE 1:** Advantages and disadvantages of various NAFLD treatment modalities.

Treatment modalities	Advantages	Disadvantages	References
Bariatric surgery	Reduce weight and appetite Short treatment period and significant therapeutic effect	Bariatric surgery may cause complications such as postoperative malnutrition and intestinal dyskinesia. These complications are largely manageable with comprehensive postoperative care. In addition, liver failure may occur in rare cases, making careful patient selection and postoperative monitoring crucial.	Nguyen and Varela, 2017; Lefere et al., 2021; Geerts and Lefere, 2023
Endoscopic therapy	Similar to bariatric surgery Less invasive and improves other components of metabolic syndrome, including blood pressure and diabetes	Similar to bariatric surgery, it is an invasive operation and is not tolerated by special patients such as the elderly	Mandour et al., 2022; Igi et al., 2024
Glucagon-like peptide 1 receptor agonists	Reduce blood sugar and weight Improve liver fibrosis Suitable for patients with co-morbid type 2 diabetes	Susceptible to hypoglycemic symptoms and adverse gastrointestinal effects such as nausea and vomiting	Lee and Kim, 2023
Statins	Improve cholesterol buildup and reduce cardiovascular disease risk Prevent hepatocellular carcinoma associated with NAFLD/NASH	Adverse reactions such as liver function abnormalities, myalgia, myositis and rhabdomyolysis may occur during treatment	Azemawah et al., 2019; Doumas et al., 2019
Vitamin E	Reduces damage to liver cells from oxidative stress and attenuates liver injury	No significant change in liver fibrosis Associated with increased risk of prostate cancer	Hadi et al., 2018
Fecal microbiota transplantation	Repair of intestinal dysbiosis and flora deficiency Wide range of clinical treatments Long-lasting therapeutic effect, not easy to recur	Due to the operation is easy to cause intestinal mucosal damage, infection and inflammation	Smits et al., 2013
*Bacteroides*, a single-strain probiotic supplementation	Maintain the balance of gut microbiota Enhance intestinal barrier function Higher safety and wider range of applications Fewer adverse effects	Gastrointestinal symptoms such as abdominal pain and diarrhea	Carpi et al., 2022; Li et al., 2024

### Reduced abundance of *Bacteroides* in NAFLD

Multiple studies have revealed the therapeutic impact of *Bacteroides* in metabolic disorders, including obesity and T2DM (Yoshida et al., 2021; Chen Q. et al., 2022; Li Y. et al., 2022). Subsequent investigations into the gut microbiota have demonstrated that augmenting the abundance of *Bacteroides* in the intestinal tract can effectively mitigate the spectrum of pathological manifestations associated with NAFLD (Li et al., 2019; Liu et al., 2019). Consequently, the correlation between *Bacteroides* and NAFLD has emerged as a focal point of scientific inquiry.

Several researchers have reported an inverse correlation between *Bacteroides* and indicators of body weight, such as the waistline and body mass index, as well as serum lipids levels, including low-density lipoprotein, total cholesterol, and triglycerides (Zeng et al., 2019; Romualdo et al., 2022; Lin et al., 2023). However, the results of current clinical research on changes in the abundance of *Bacteroides* in NAFLD patients are conflicting. One clinical study found that the abundance of *Bacteroides* increased as NAFLD pathology progressed, indicating a potential association between *Bacteroides* and NAFLD severity (Boursier et al., 2016). Conversely, a study on obese patients with NAFLD showed a significant reduction in *Bacteroides* abundance (Jin and Xu, 2023). In another study, compared to 28 healthy controls, 39 patients with biopsy-proven NAFLD had a prominently lower abundance of *Bacteroides* (Da Silva et al., 2018). Similar trends were observed in children with NAFLD, whereby one study demonstrated a decrease in *Bacteroides* abundance compared to in the controls (Del Chierico et al., 2017). Additionally, a recent study using a mouse model of NAFLD induced by a Western diet found an inverse correlation between *Bacteroides* abundance and the progression of NAFLD (Romualdo et al., 2022). Overall, these findings support the notion of a reduced abundance of *Bacteroides* in patients with NAFLD.

### Therapeutic role of *Bacteroides* in NAFLD

In healthy adults, *Bacteroides* constitute 20–80% of the gut microbiota, playing a crucial role as the dominant flora in the gut (Tan et al., 2019a). As prominent commensals and mutualists in the human gut, *Bacteroides* species have adapted to the gut and established a solid association with the human host (Wexler and Goodman, 2017). *Bacteroides* spp. possess polysaccharide utilization loci that enable them to metabolize polysaccharides that are not easily absorbed in the intestine, providing energy to adjacent bacteria (Zafar and Saier, 2021). This process helps maintain the balance of the gut microbiota. *Bacteroides* have attracted significant attention from scientists due to their remarkable adaptive properties, particularly their potential benefits.

In recent years, various effective drugs, such as glucose-lowering drugs, antioxidants, lipid-lowering agents, and natural bile acid treatments, have been used to treat NAFLD (Mantovani and Dalbeni, 2021). Interestingly, gut microbiota analysis has shown an increase in *Bacteroides* abundance after treatment with Raw Bowl Tea polyphenols, atorvastatin, *Ganoderma* meroterpene derivative, and the prebiotic inulin for NAFLD (Liu et al., 2019; Qiao et al., 2020; Aoki et al., 2021; Li H. et al., 2022). For instance, atorvastatin was found to reduce lipid levels, improve HFD-induced hepatic steatosis, and increase *Bacteroides* abundance (Li H. et al., 2022). In fa/fa rats, the oral administration of *Ganoderma* meroterpene derivative was shown to reduce *de novo* lipogenesis, reverse non-alcoholic hepatic steatosis, and enhance *Bacteroides* abundance (Qiao et al., 2020). Furthermore, the knockout of sirtuin 2, which has a preventive role against NAFLD, was associated with a decreased abundance of *Bacteroides* and aggravation of NAFLD (Li et al., 2023). *Bacteroides* has also demonstrated potential for treating ethanol- or drug-induced liver damage in several studies (Sangineto et al., 2022; Wang H. et al., 2022). These findings suggest that *Bacteroides* might have a therapeutic role against NAFLD.

### Effects of *Bacteroides* on the liver

Non-alcoholic fatty liver disease (NAFLD) is characterized by histopathologic abnormalities in the liver, including lipid deposition, steatosis, hepatic balloon degeneration, and inflammation, which can lead to liver fibrosis (Marchisello et al., 2019). *Bacteroides* species have shown excellent metabolic regulation. Recent studies strongly indicate that administering *Bacteroides uniformis* (*B. uniformis*), *Bacteroides vulgatus* (*B. vulgatus*), *Bacteroides acidifaciens* (*B. acidifaciens*) and *Bacteroides thetaiotaomicron* (*B. thetaiotaomicron*) can alleviate various metabolic disorders, such as insulin resistance, adiposity, and steatosis, which are the main manifestations of metabolic disorders in NAFLD mice (Gauffin Cano et al., 2012; Yang et al., 2017; Lee et al., 2021; Xu et al., 2023; Li et al., 2024). Importantly, *Bacteroides* has also been shown to reduce liver inflammation by enhancing the immune function and to contribute to the alleviation of liver fibrosis (Fernández-Murga and Sanz, 2016; López-Almela et al., 2021).

### Effects of *Bacteroides* on the gut

Changes in gut microbiota and the gut barrier function can influence the development and progression of NAFLD, leading to elevated levels of endotoxin and increased permeability to bacterial endotoxins (Jin et al., 2016). However, numerous studies have demonstrated that *Bacteroides* can improve gut homeostasis by ameliorating gut microbiota disorders and regulating the tight-junction (TJ) proteins (El Kaoutari et al., 2013; Lopez-Siles et al., 2017; Nagpal et al., 2018; López-Almela et al., 2021). Moreover, *Bacteroides* influence the levels of various metabolites, including short-chain fatty acids (SCFAs), branched-chain amino acids (BCAAs), and taurine, that are key for the absorption of lipid-soluble foods, maintaining the integrity of the gut barrier to prevent the translocation of bacteria, and regulating glucolipid metabolism. These metabolites have emerged as crucial factors in attempts at regulation of the pathological processes of NAFLD (Ji et al., 2019; Yoshida et al., 2021; Xu et al., 2023; Zhou et al., 2023). Based on the available evidence, some *Bacteroides* strain may alleviate NAFLD. Additional details are provided in Table 2.

**TABLE 2:** *In vivo* evidence for *Bacteroides* Strain in the treatment of NAFLD.

*Bacteroides* Strain	Subject	Dosage	Treatment period	Results measures	Conclusion	References
*B. uniformis* CECT 7771	Male C57BL/6 mice	5.0 × 10^8^ CFU/day	7 Weeks	Body weight Adipose tissue weight Biochemical parameters Hepatic steatosis Adipocyte size Macrophage functionality Dendritic cell functionality	*B. uniformis* CECT 7771 ameliorates metabolic disorders and immune dysfunction associated with HFD-induced gut flora dysbiosis.	Gauffin Cano et al., 2012
*B. acidifaciens* JCM10556	C57BL/6, CD11c-Cre, Villine-Cre, LysM-Cre, and Atg7f/f mice	5 × 10^9^ CFU/day	10 Weeks	Body weight and fat weight Blood glucose and insulin Dipeptidyl peptidase-4 Glucagon-like peptide-1	*B. acidifaciens* JCM10556 has the ability to treat metabolic diseases such as diabetes and obesity.	Yang et al., 2017
*B. uniformis* CBA7346	Male C57BL/6J mice	1 × 10^6^ CFU thrice a week	12 Weeks	Body and liver weights Serum lipids Liver injury and steatosis Hepatic lipid metabolism	*B. uniformis* CBA7346 improves insulin resistance and modulates adipogenesis in HFD-induced NAFLD mice.	Lee et al., 2021
*B. vulgatus* Bv46	Female SD rats	2 × 10^9^ CFU every other day	6 Weeks	Body and liver weights Serum lipids Food intake Serum inflammation factor	*B. vulgatus* Bv46 reduces systemic inflammation, promoted cholesterol excretion, and ameliorated lipid disorders induced by HFD.	Xu et al., 2023
*B. uniformis* CECT 7771	Male C57BL/6J mice	5 × 10^7^ CFU/day	17 Weeks	Body weight Epididymal fat Insulin-dependent metabolic routes Visceral fat thermogenesis Inflammation	*B. uniformis* CECT 7771 combined with wheat bran extract alleviates metabolic disorders and immune dysregulation in obese mice.	López-Almela et al., 2021
*B. thetaiotaomicron* ATCC 29148	Male C57BL/6J mice	1 × 10^8^ CFU thrice a week	12 Weeks	Body weight Serum lipids Insulin Hepatic steatohepatitis and liver injury	*B. thetaiotaomicron* ATCC 29148 promotes gut-liver folate metabolism and increases the proportion of unsaturated fatty acids to improve NAFLD.	Li et al., 2024
*B. dorei* DSM17855 and *B. vulgatus* ATCC8482	Male C57BL/6J mice	2.5 × 10^9^ CFU *B. dorei* and *B. vulgatus mix* five times a week	12 Weeks	Body weight Brown adipose tissue weight Plasma branched-chain Amino acids Blood glucose	*B. dorei* DSM17855 and *B. vulgatus* ATCC8482 inhibit obesity by enhancing branched-chain amino acid catabolism in brown adipose tissue.	Yoshida et al., 2021
*B. vulgatus* 8482 and *B. dorei* 17855	C57BL/6 mice	2.5 × 10^9^ CFU *B. dorei* and *B. vulgatus* mix five times a week	10 weeks	Endotoxemia and systemic inflammation LPS production Tight-junction formation Intestinal immune response	*B. vulgatus* 8482 and *B. dorei* 17855 may improve the intestinal environment, reduce intestinal microbial lipopolysaccharide production, and improve endotoxemia.	Yoshida et al., 2018
*B. fragilis* ZY-312	C57BL/6 mice STAT3 conditional gene knockout mice	1 × 10^9^ CFU/day	15 days	Intestinal length shortening Intestinal injury Stem cell and goblet cell regeneration STAT3 signaling pathway	*B. fragilis* ZY-312 attenuates intestinal injury by activating the STAT3 signaling pathway to promote intestinal barrier integrity.	Zhou et al., 2022
*B. fragilis* HCK-B3 and *B. ovatus* ELH-B2	Female C57BL/6J mice	1 × 10^9^ CFU/day	5 days	Treg/Th-17 balance TNF-α IL-10 NF-κB	*B. fragilis* HCK-B3 and *B. ovatus* ELH-B2 regulate cytokine production and restore Treg/Th-17 balance to alleviate intestinal inflammation.	Tan et al., 2019b

The results presented in the table demonstrate statistical significance. *B. uniformis*, *Bacteroides uniformis*; *B. acidifaciens*, *Bacteroides acidifaciens*; *B. vulgatus*, *Bacteroides vulgatus*; *B. thetaiotaomicron*, *Bacteroides thetaiotaomicron*; *B. dorei*, *Bacteroides dorei*; *B. fragilis*, *Bacteroides fragilis*; *B. ovatus*, *Bacteroides ovatus*; CFU, colony forming units.

### Underlying mechanisms of *Bacteroides*’ action in NAFLD

**FIGURE 1:**
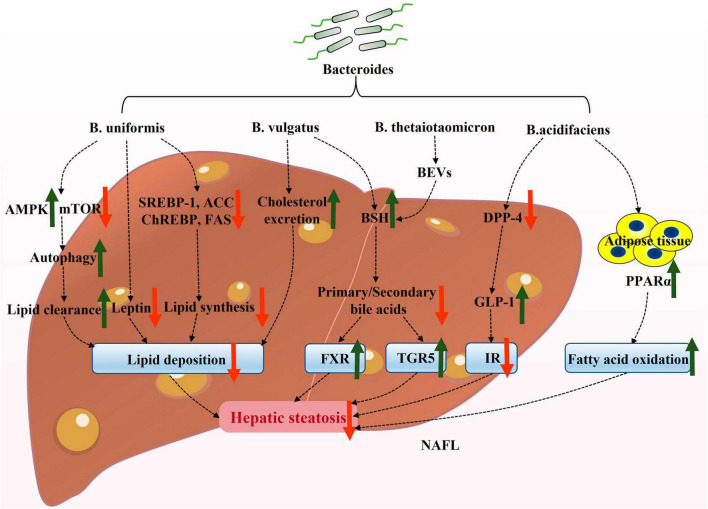
Potential mechanisms underlying the attenuation of hepatic steatosis by *Bacteroides* in NAFLD. *Bacteroides* and its active ingredient, bacterial extracellular vesicles (BEVs), alleviate hepatic steatosis and associated insulin resistance. AMPK, adenosine monophosphate-activated protein kinase; mTOR, mechanistic target of rapamycin; SREBP-1, sterol regulatory element-binding protein-1; ChREBP, carbohydrate response element-binding protein; ACC, acetyl-CoA carboxylase; FAS, fatty acid synthase; BEVs, bacterial extracellular vesicles; BSH, bile salt hydrolase; TGR5, takeda G protein-coupled receptor 5; FXR, farnesoid X receptor; DPP-4, dipeptidyl peptidase-4; GLP-1, glucagon-like peptide-1; IR, insulin resistance; PPARα, peroxisome proliferator-activated receptor α.

**FIGURE 2:**
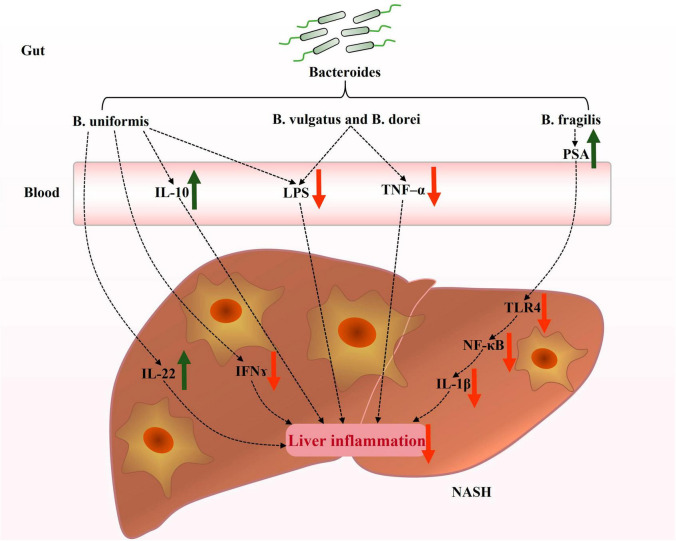
Potential mechanisms contributing to the prevention of hepatic inflammation by *Bacteroides* in NAFLD. *Bacteroides* regulated inflammation and immune response in the livers of NAFLD patients. The current focus on the mechanisms underlying the improvement of hepatic inflammation by *Bacteroides* primarily revolves around the production of inflammatory factors and LPS and TLR4 signaling. IL-22, interleukin-22; IFN-γ, interferon-γ; IL-10, interleukin-10; LPS, lipopolysaccharide; TNF-α, tumor necrosis factor-α; PSA, polysaccharide A; TLR4, toll-like receptor 4; NF-κB, nuclear factor-κB; IL-1β, interleukin-1β.

**FIGURE 3:**
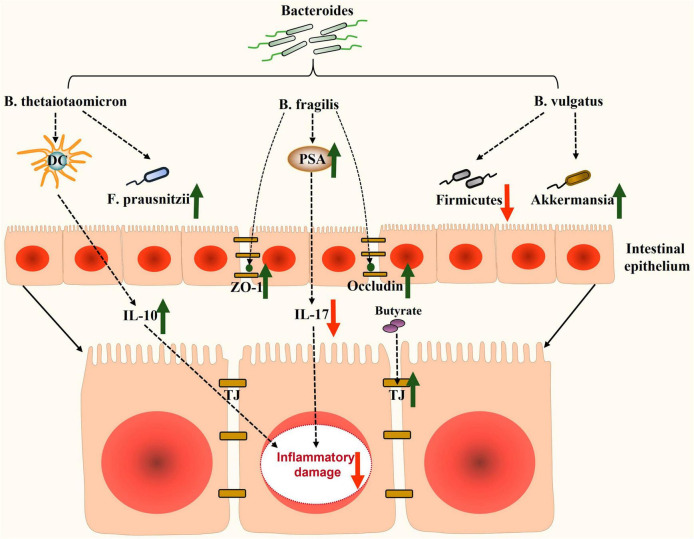
Potential mechanisms involved in enhancing the intestinal barrier function by *Bacteroides* in NAFLD. *Bacteroides* and its metabolites enhance the barrier function of the intestine through modulating intestinal inflammatory factors, improving the expression of tight-junction proteins, and restoring intestinal dysbiosis. DC, dendritic cells; *F. prausnitzii*, *Faecalibacterium prausnitzii*; IL-10, interleukin-10; ZO-1, zonula occludens-1; PSA, polysaccharide A; IL-17, interleukin 17; TJ, tight-junction.

In recent years, there has been significant research on the role of *Bacteroides* in NAFLD. However, the precise mechanisms by which *Bacteroides* act remain unclear. NAFLD is strongly associated with hepatic steatosis, inflammation, and intestinal dysfunction. Existing evidence suggests that *Bacteroides* can prevent NAFLD by mitigating hepatic steatosis (Figure 1) and liver inflammation (Figure 2), and enhancing the intestinal barrier function (Figure 3).

### Hepatic steatosis and *Bacteroides*

The excessive deposition of free fatty acids is a major contributor to the initiation of NAFLD. The excessive accumulation of free fatty acids in the liver promotes the progression from hepatic steatosis to NASH and cirrhosis through the generation of reactive oxygen species (Li et al., 2019). Interestingly, the transition from hepatic steatosis to inflammation is triggered by imbalances in the adipocytokines and lipotoxicity (Khan and Newsome, 2018). Therefore, effective treatment for NAFLD involves improving steatosis and its associated metabolic dysfunction.

### Reducing lipid deposition

Liver lipid deposition is primarily caused by excessive fat production or impaired fatty acid oxidation (FAO) (Ipsen et al., 2018). *De novo* lipogenesis refers to the process of synthesizing saturated fatty acids from acetyl coenzyme A, derived from glycolysis (Ipsen et al., 2018). This process is regulated by multiple transcription factors, such as sterol regulatory element-binding protein-1 (SREBP-1) activated by insulin, and carbohydrate response element-binding protein (ChREBP) activated by glucose, which are critical regulators of hepatic lipogenesis (Sanders and Griffin, 2016). SREBP-1 and ChREBP also regulate the expression of hepatic lipid synthesis-related genes, including acetyl-CoA carboxylase (ACC), stearoyl-CoA desaturase 1 (SCD1), and fatty acid synthase (FAS) (Postic et al., 2007; Mu et al., 2020).

In obese ob/ob mice, the expressions of SREBP-1c and ChREBP were found to be significantly increased in the liver, and it was reported that reducing the levels of either factor could contribute to the attenuation of hepatic steatosis (Dentin et al., 2006). In a mouse model of HFD-induced NAFLD, supplementation with *B. uniformis* significantly downregulated the expression of hepatic lipogenesis-related proteins, such as SREBP-1, ChREBP, ACC, FAS, and leptin; thereby alleviating the progression of NAFLD (Lee et al., 2021). Another study also supported this point (López-Almela et al., 2021). In rats with metabolic dysfunction induced by HFD, the oral administration of *B. vulgatus* significantly reduced body weight gain, improved the lipid profile, promoted cholesterol excretion, and enhanced lipid homeostasis (Xu et al., 2023).

*Bacteroides* also appear to actively contribute to FAO. Peroxisome proliferator-activated receptor α (PPARα) is a key receptor involved in FAO, and its activation has been shown to reduce hepatic steatosis in obese ob/ob mice (Gao et al., 2015). Interestingly, research found that *B. acidifaciens* prevented obesity in mice by upregulating PPARα (Yang et al., 2017). Additionally, HFD-induced mice treated with *B. uniformis* showed an increased activation of adenosine monophosphate-activated protein kinase (AMPK) and a reduced expression of the mechanistic target of rapamycin (mTOR) (Lee et al., 2021). Yan et al. (2022) found that the activation of AMPK and the inactivation of mTOR initiated autophagy to clear hepatic lipids. Therefore, it was hypothesized that *B. uniformis* may induce autophagy through the AMPK/mTOR signaling pathway to regulate lipid homeostasis, thus promoting fat removal.

*Bacteroides* have been found to significantly modulate lipid metabolism in animal models (Lee et al., 2021; López-Almela et al., 2021; Li H. et al., 2022; Xu et al., 2023). These studies indicate that *Bacteroides* ultimately reduce the accumulation of fat in the liver via inhibiting SREBP-1 and ChREBP expression, as well as related adipogenic genes, and by promoting FAO-related pathways. This suggests that several *Bacteroides* spp., such as *B. uniformis*, *B. vulgatus*, and *B. acidifaciens*, play a critical role in reducing lipid deposition.

### Improving insulin resistance

Insulin resistance is a key factor in the development of NAFLD. Impaired insulin signaling in adipose tissue leads to excessive fatty acids transport to the liver via dysregulated lipolysis, contributing to the development of NASH (Lomonaco et al., 2012; Lian et al., 2020). Insulin-dependent lipogenesis is another crucial mechanism in NAFLD (Smith et al., 2020). Studies have demonstrated the protective role of *Bacteroides* against insulin resistance related to steatosis and the accumulation of lipids in the liver. Some studies have shown that the beneficial effects of *Bacteroides* on insulin resistance were partly due to increased insulin levels in the body or the alleviation of metabolic endotoxemia (Rao et al., 2016; Lee et al., 2021).

In 2017, a study conducted by Yang et al. (2017) demonstrated that the oral administration of *B. acidifaciens* resulted in decreased blood glucose levels and increased insulin levels in mice fed a HFD. Subsequently, the authors further investigated the effect of *B. acidifaciens* in regulating insulin resistance. Importantly, the oral administration of *B. acidifaciens* or its culture supernatant led to a reduction in the levels of dipeptidyl peptidase-4 (DPP-4) in the ileum of the small intestine (Yang et al., 2017). DPP-4 is an enzyme known for degrading GLP-1. These findings suggest that *B. acidifaciens* may inhibit the activity of the DPP-4 enzyme, leading to the activation of GLP-1 and a subsequent improvement in glucose tolerance and insulin sensitivity. This evidence strongly suggests that *B. acidifaciens* may have the potential to alleviate hepatic steatosis by improving insulin resistance.

### Regulating bile acid metabolism

*Bacteroides* spp. are prominent Gram-negative bacteria that produce bacterial extracellular vesicles (BEVs), a novel secretion system capable of transporting a wide range of chemically diverse cargoes, including lipids, membrane-embedded and associated proteins, peptidoglycan, and nucleic acids (Perez-Cruz et al., 2015; Rakoff-Nahoum et al., 2016; Cecil et al., 2019; McMillan and Kuehn, 2021). Studies have revealed that BEVs produced by *B. thetaiotaomicron* in the gastrointestinal tract were enriched with specific proteins and enzymes, such as dipeptidyl peptidases, bile salt hydrolase (BSH), and asparaginase, thus influencing the host cell’s biosynthetic pathways (Stentz et al., 2022). BSH activity is a gut microbial process that significantly impacts the host’s local (gastrointestinal) and systemic (hepatic) functions (Joyce et al., 2014b). Controlled experimental systems indicated that bacterial BSH activity had a profound effect on the host bile acid profiles, improving conditions like hypercholesterolemia and obesity, thus serving as a crucial mechanism by which microbiota regulate the host lipid metabolism (Joyce et al., 2014a).

*Bacteroides vulgatus* has been shown to exhibit BSH activity (Xu et al., 2023). BSH catalyzes the deconjugation of bile acids (BAs), liberating primary BAs and glycine/taurine moieties (Horáčková et al., 2018). Coincidentally, taurine, produced through deconjugation, has also been reported to play a role in physiological processes, such as antioxidant defense, immune regulation, and lipid homeostasis, thereby blunting the progression of NAFLD (Lambert et al., 2015; Kim et al., 2022). Subsequently, a portion of the primary BAs is affected by the gut microbiota through processes like dehydroxylation, dehydrogenation, and sulfation, resulting in the production of secondary BAs (Foley et al., 2019). An imbalance in the ratio of primary to secondary bile acids is a significant factor in accelerating NAFLD. The BSH enzyme produced from *B. vulgatus* restores this imbalance and regulates the host metabolism by activating farnesoid X receptor (FXR) and takeda G protein-coupled receptor 5 (TGR5) (Arab et al., 2017; Jiao et al., 2018; Pathak et al., 2018). As mentioned earlier, the ratio of primary to secondary bile acids is regulated by BSH. Therefore, we hypothesize that *Bacteroides* activate the expression of FXR and TGR5 by modulating the bile acids ratio through BSH activity, and by reducing hepatic steatosis.

Collectively, current evidence confirms the role of *Bacteroides* and its components in metabolic dysfunction associated with steatosis (Figure 1), which may partially explain the mechanism of action of *Bacteroides* in improving NAFLD.

### Liver inflammation and *Bacteroides*

NAFLD is a manifestation of liver-related metabolic syndrome. Numerous studies have demonstrated there are alterations of the immune microenvironment during the development of NAFLD (Ilan, 2013; Huby and Gautier, 2022). The gut microbiota plays various roles in the intestinal barrier function, immunity, and metabolism by mediating different forms of host responses. The gut microbiota with its various metabolites influence immune homeostasis and liver metabolism, thereby impacting the progression of NAFLD (Kolodziejczyk et al., 2019; D’Alessandro et al., 2020). An association between low levels of *Bacteroides* and liver damage in alcoholic fatty liver disease has also been established (Ferrere et al., 2017). Further research on *Bacteroides* has revealed its essential role in regulating hepatic inflammation in NAFLD.

### Regulating cytokines in the liver

Interferon-γ (IFN-γ) and tumor necrosis factor-α (TNF-α) are recognized as pro-inflammatory cytokines that play crucial roles in the pathophysiology of NAFLD. These factors recruit inflammatory cells and/or activate macrophages, leading to a hepatic inflammatory response (Tilg, 2010; Li et al., 2021). Importantly, one study found that mice treated with *B. vulgatus* and *Bacteroides dorei* (*B. dorei*) exhibited significantly reduced serum TNF-α levels (Yoshida et al., 2018). In obese mice, *B. uniformis* increased the expression of anti-inflammatory interleukin-22 (IL-22) and decreased the production of pro-inflammatory IFN associated with high-fat, high-sugar diet-induced liver damage, contributing to a greater balance of the hepatic immune status (López-Almela et al., 2021). Notably, IL-22 has also shown a protective response to liver tissue injury, attenuating the inflammatory response in NAFLD models, and also alleviating oxidative stress, endoplasmic reticulum stress, and apoptosis (Zai et al., 2021; Abdelnabi et al., 2022).

Furthermore, *Bacteroides* also exert a protective effect on the liver. The administration of *B. uniformis* increased interleukin-10 (IL-10) levels in mice fed a HFD (Gauffin Cano et al., 2012). Several studies have found an association between NAFLD severity and serum IL-10 levels reduction, suggesting the involvement of IL-10 in immune regulation in the liver (den Boer et al., 2006; Paredes-Turrubiarte et al., 2016). These findings suggest that *Bacteroides* modulate the hepatic cytokines related to immune regulation (such as TNF-α, IL-22, IFN-γ, and IL-10) and downregulate the hepatic inflammatory responses (Figure 2).

### Inhibiting gut-derived LPS and TLR4 signaling

Lipopolysaccharide (LPS), a typical pathogen-associated molecular pattern, mediates inflammatory injury in hepatocytes and has been found to induce inflammatory liver injury through the downstream toll-like receptor 4 (TLR4) signaling pathway (Qi et al., 2020; An et al., 2022). LPS is a crucial factor in the induction of inflammatory responses in the liver and is expressed abundantly in patients with NASH (Carpino et al., 2020). Treatment with *B. vulgatus* and *B. dorei* has been reported to reduce circulating LPS levels via strengthening the gut barrier function (Yoshida et al., 2018). Additionally, *B. uniformis* reduces the expression of acyl carrier protein, a key component required for LPS biosynthesis (Wang et al., 2021b). Polysaccharide A (PSA), an immunogenic component of *B. fragilis* capsules, inhibited the TLR4/nuclear factor-κB (NF-κB) signaling pathway and suppressed the downstream inflammatory factor interleukin-1β (IL-1β) in a model of LPS-induced liver inflammation (Wang et al., 2021c). These findings suggest that the potential mechanism by which *Bacteroides* attenuate liver inflammation is by modulating LPS and TLR4 signaling in NAFLD (Figure 2).

In a safety assessment, *B. uniformis* reduced inducible nitric oxide synthase (iNOS) expression in the liver, which was increased in the livers of immunosuppressed mice (Fernández-Murga and Sanz, 2016). iNOS was demonstrated to be one of the key enzymes involved in producing nitric oxide (NO) from the amino acid L-arginine, while excessive NO production has been associated with the etiology of many liver diseases, such as liver fibrosis (Iwakiri, 2015). This finding indicated that *Bacteroides* might possess protective abilities against liver fibrosis. However, further studies are still needed to confirm *Bacteroides*’ effect on NAFLD progression to fibrosis and its exact functional mechanism.

### Intestinal barrier function and *Bacteroides*

The gut–liver axis facilitates bidirectional communication between the biliary tract, portal vein, and systemic circulation. The intestinal barrier serves as the first line of defense against endotoxin-induced liver damage. Disruption of the intestinal barrier function, caused by factors such as intestinal barrier damage, inflammation, or microbial imbalances, can lead to increased intestinal permeability. This can allow endotoxins to translocate into the bloodstream and disseminate to the liver, thereby promoting the progression of NAFLD to NASH (Wigg et al., 2001; Mouries et al., 2019; Hrncir et al., 2021). Several theories support the protective effect of *Bacteroides* in maintaining the integrity of the intestinal barrier.

### Promoting tight-junctions in the gut

The intestinal epithelial barrier function relies on the TJs of intestinal epithelial cells. These TJs consist of various tight-junction proteins, including zonula occludens-1 (ZO-1) and occludin, which play crucial roles in regulating intestinal permeability (Wigg et al., 2001). *B. fragilis*, for instance, promotes the expression of TJ proteins, such as ZO-1 and occludin (Wang et al., 2021a; Zhou et al., 2022). *Bacteroides* spp. also promote the fermentation of polysaccharides in the intestine, resulting in the production of SCFAs, like acetate, propionate, and butyrate (El Kaoutari et al., 2013). Butyrate, in particular, helps maintain the intestinal barrier function by providing essential energy to the epithelium and upregulating TJ protein expression (Donohoe et al., 2011; Nagpal et al., 2018). Additionally, butyrate exhibits anti-inflammatory, metabolic modulating, and antioxidant effects that contribute to the amelioration of NAFLD (Amiri et al., 2022).

*Bacteroides* also contribute to preservation of the mucus and microbial barrier of the gut. *B. thetaiotaomicron*, for example, maintains the function of the intestinal mucus barrier by inducing the expression of the mucin genes *muc2* and *muc4* and by increasing the number of goblet cells (Wrzosek et al., 2013). Moreover, *Bacteroides* play a role in constructing a biological barrier that protects the host from pathogens, like LPS-producing *Proteobacteria* (Wang L. et al., 2022).

### Alleviating inflammatory damage in the gut

*Bacteroides* also play a significant role in maintaining gut immune homeostasis, including the regulation of IL-10 as well as T helper cell 17 (Th17). For instance, *B. thetaiotaomicron* promotes the response of mucosal dendritic cells and contributes to the expression of IL-10 levels (Durant et al., 2020; Fonseca et al., 2022). Research has shown that *B. fragilis* PSA suppressed the pro-inflammatory response, thus preventing colitis by inhibiting Th17 cells (Troy and Kasper, 2010). Th17 cells are known to secrete pro-inflammatory cytokines, such as interleukin 17 (IL-17), interleukin 21 (IL-21), and TNF-α (Chen and O’Shea, 2008). Interestingly, IL-17 significantly contributes to the major comorbidity of NAFLD, specifically early atherosclerosis, which was confirmed in a study involving obese NAFLD patients (Tarantino et al., 2014). Furthermore, studies have demonstrated that *Bacteroides ovatus* (*B. ovatus*) and *B. fragilis* alleviate LPS-related intestinal inflammation by restoring Treg/Th17 homeostasis or regulating cytokine production (Tan et al., 2019b). Additionally, SCFAs inhibited the NF-κB pathway and LPS-induced NOD-like receptor family pyrin domain containing 3 (NLRP3) activity, thereby reducing intestinal inflammatory injury (Feng et al., 2018).

### Restoring the gut microbiota homeostasis

Long-term consumption of a HFD disrupts the balance of the gut microbiota, promoting the progression of NAFLD. Restoring gut microbiota homeostasis has been identified as a key target for NAFLD treatment, and various therapeutic modalities, such as fecal microbial transplantation and next-generation probiotics, have been proposed as treatments (Rotman and Sanyal, 2017; Campagnoli et al., 2022; Riezu-Boj et al., 2022; Maestri et al., 2023; Singh et al., 2023). *Bacteroides* play a crucial role in regulating gut microbiota homeostasis.

*Firmicutes* have been reported to be associated with the acquisition of more energy (Turnbaugh et al., 2006). An increase in *Firmicute*s was found to be one of the identified features of the gut microbiome in NAFLD model mice (Romualdo et al., 2022). *B. vulgatus* was found to decrease the levels of *Firmicutes* at the phylum level and reshape the imbalanced gut microbiota induced by HFD (Xu et al., 2023). In mice with obesity, the administration of *B. uniformis* effectively restored the gut microbiota induced by a high-fat, high-sugar diet (López-Almela et al., 2021). Another study showed that *Bacteroides* participated in the production of BCAAs in the colon (Diether and Willing, 2019), which promoted beneficial intestinal bacteria growth, further enhancing the protective effect of the intestinal barrier (Zhou et al., 2018).

In a study conducted in 2023, researchers discovered that *Faecalibacterium prausnitzii* (*F. prausnitzii*) exhibited a beneficial effect on liver inflammation and hepatic steatosis in NASH mice (Shin et al., 2023). It was observed that *F. prausnitzii* may engage in a mutually beneficial relationship with certain *Bacteroides* species. Heinken et al. (2014) demonstrated that the production of acetate by *B. thetaiotaomicron* facilitated the growth of *F. prausnitzii*. Furthermore, *B. thetaiotaomicron* is capable of fermenting pectin to release pectin derivatives that can be utilized by *F. prausnitzii* (Lopez-Siles et al., 2017). Interestingly, You et al. (2023) revealed that *B. vulgatus* restored the depletion of *Akkermansia* induced by a HFD. *Akkermansia*, known for its ability to improve metabolic disorders in humans, is negatively impacted by mucin deficiency (Van Herreweghen et al., 2018). The study by You et al. (2023) further demonstrated that a metabolite called N-acetyl-D-glucosamine (GlcNAc), induced by *B. vulgatus*, had a mucin-stimulating effect, effectively reversing the decline of *Akkermansia* in mice fed a HFD. Additionally, *B. vulgatus* serves as a provider of nutrients, supplying microbiota-accessible carbohydrates, such as mucin glycans, to maintain microbial homeostasis, particularly with *Lactobacilli* and *Bifidobacteria*, which play a crucial role in the mucus integrity, indirectly reinforcing the cooperative network with *Akkermansia* (You et al., 2023; Zhang et al., 2023). Therefore, it can be inferred that the therapeutic effects of *Bacteroides* in NAFLD are partially mediated by increasing the abundance of beneficial flora in the gut. In conclusion, *Bacteroides* contribute to the enhancement of the intestinal barrier function by modulating the epithelial, mucosal, and immune components, as well as the microbial ecosystem (Figure 3).

## Conclusion and future perspectives

Recent findings have unveiled that gut microbiota dysbiosis serves as a risk factor that can exert an influence on and contribute to the pathogenesis of NAFLD. Based on the aforementioned *in vivo* studies, *Bacteroides* not only rectifies the dysbiosis of the gut microbiota but also mitigates NAFLD by modulating metabolic and inflammatory factors, while enhancing intestinal barrier functions. Consequently, *Bacteroides* exhibits the potential to be a novel therapeutic modality for NAFLD.

*Bacteroides* has been discovered to possess a remarkable ameliorating effect on NAFLD, thus paving the way for its prospective application in clinical settings. Reasonable assumptions regarding the clinical utilization of *Bacteroides* have been formulated based on animal experiments and clinical studies involving other probiotics. For the recommended clinical dosage of *Bacteroides*, we hypothesized that a daily dose ranging from 2 × 10^8^ to 1 × 10^10^ colony forming units (CFU) (Silva-Sperb et al., 2019; Laue et al., 2023) is suitable for healthy adults. This dosage range is considered safe and effective. Similar to most probiotic delivery methods, *Bacteroides* can be administered through colony transplantation, oral formulations, injections, and enteric capsules. Among these modalities, we favor the use of enterosoluble capsules, as they not only protect the flora from gastric acid but also enhance portability, thereby improving patient adherence. In summary, *Bacteroides* is deemed beneficial, however, potential risks may arise, such as exacerbation of intestinal flora disorders leading to abdominal pain and diarrhea as well as allergic reactions triggered by sensitivities to specific ingredients.

There are still some questions that need to be addressed regarding the involvement of *Bacteroides* in the regulation of NAFLD. First, although the immune regulatory effects of *Bacteroides* in the liver have been demonstrated, the specific mechanisms by which *Bacteroides* and its active ingredients participate in immune status regulation in the liver have yet to be elucidated and require further exploration in future studies. Second, *Bacteroides* encompass numerous species, including *B. uniformis*, *B. fragilis*, *B. vulgatus*, *B. thetaiotaomicron*, and others mentioned in the text. These species play distinct roles in various aspects of NAFLD development, and it remains unclear whether their combined use would yield superior outcomes. The combination of probiotics is widely employed in clinical practice. Therefore, future experiments could employ germ-free mice to evaluate the effects of combinations of flora. Last, the current experimental findings are primarily based on *in vitro* studies or animal models, thus lacking substantial clinical evidence. By constructing relevant clinical cohorts, clinical studies combined with fecal microbiota transplantation could be conducted to validate the efficacy and safety of *Bacteroides* in human NAFLD treatment.

In summary, *Bacteroides* and its metabolism play a therapeutic role in NAFLD by ameliorating hepatic steatosis, insulin resistance, and dysbiosis. More clinical studies in the future will promote the clinical application of *Bacteroides* in NAFLD.

## Author contributions

JuZ: Writing – original draft, Writing – review & editing. JiZ: Investigation, Writing – review & editing. ZH: Investigation, Writing – review & editing. HL: Conceptualization, Funding acquisition, Writing – review & editing.
